# Syndemic analysis of the pandemic COVID-19 crisis: a multidisciplinary “Understand—Anticipate – Propose” meta-method

**DOI:** 10.1186/s13690-022-00951-x

**Published:** 2022-08-19

**Authors:** Didier Lepelletier, Camille Souhard, Christian Chidiac, Franck Chauvin, Zeina Mansour

**Affiliations:** 1 High Council for Public Health, Ministry of Health, 10 place des cinq martyrs du lycée Buffon, 75015 Paris, France; 2 Hospital Hygiene Department, Nantes University Hospital, Nantes, France; 3 UR 1155 – Nantes UniversityIICiMed, Institut de Recherche en Santé 2, 44200 Nantes, France; 4 Department of Infectious and Tropical Diseases, Lyon University Hospital, Lyon, France; 5 CIRI équipe PH3ID - INSERM - U1111- Université Claude Bernard Lyon 1 - CNRS - UMR5308 - ENS de Lyon, Lyon, France; 6 Centre Hygée, Institut Presage, University of Saint-Etienne, CIC-EC Inserm 1408, Saint-Etienne, France; 7 Regional Committee for Health Education, Provence Alpes Côte d’Azur (PACA), Marseille, France

**Keywords:** Covid-19, Pandemic, Syndemic crisis, Meta-method, Public health planning

## Abstract

**Background:**

In order to understand the pandemic COVID-19 crisis in a forward-looking way, the French High Council for public health (HCSP) has designed a conceptual scheme for public health planning based on L. Green's model in order to better understand the issues at stake, by identifying dangers and levers for action. The final aim was to establish priorities and guidelines in order to anticipate the collateral consequences of the management of the crisis and be better prepared for the next one.

**Method:**

A public health conceptual framework PRECEDE-PROCEED adapted to the Covid-19 health crisis was developed using both a graphic (concept map) and analytic (to make the conceptual scheme functional) approaches. Then, a "meta-method" was applied using three distinct cognitive stages: understanding, anticipation and proposals of action.

**Results:**

The conceptual framework was broken down into 10 technical sheets covering essential diagnoses and integrating different public health determinants. Each of these was broken down into three cognitive stages, allowing for a diagnosis of understanding, a scenario of anticipation and a strategic analysis of action according to the chronology: understand-anticipate-propose. From these 10 technical sheets, 32 guidelines have been proposed.

**Conclusion:**

This work is intended to allow reflections on public health approaches to strengthen and anticipate health crisis management and health planning by politic managers working at national or sub-national level.

## Introduction

All the determinants that have a direct or indirect impact on the evolution of the Covid-19 pandemic must be considered, particularly the lockdown consequences of the epidemic on the population's health, in line with the World Health Organization’s definition, which considers health to be a state of complete physical, mental and social well-being [1]. Considering the syndromic nature of COVID-19, as first described by Merrill Singer in the 1990s [2] and more recently by Robert Horton [3], the HCSP has adopted a broad vision that allows an integrated approach to understanding and managing a health crisis. Syndemia is characterized by the accumulation of at least two health problems in certain populations in relation to socio-economic contexts or situations, with this accumulation resulting in an aggravated state of health for these populations [4].

Combating this syndrome cannot therefore be limited to combating the SARS-CoV-2 pandemic, but must take into account the severity of risk co-factors that accumulate in certain populations.

The concept of "One Health", "one human, animal and environmental health", or more recently "global health", has been put forward since the early 2000s, with the awareness of the close links between human health and its environment [5].

It aims to promote a multidisciplinary and global approach to health issues. This concept places health in all public policies and fully justifies taking into account multiple and varied determinants.

The global approach to health implies a consensus on all the determinants of health but also on the factors influencing the evolution of a health crisis in a favorable or unfavorable way. These include direct effects on the indicators monitoring the epidemic, or indirect effects such as the medium- and long-term impact on the health status of the population as defined by the WHO [6, 7]. This approach requires broad monitoring of a wide range of indicators.

The HCSP documented the syndemic nature of the crisis in its opinion on the Covid-19 health crisis and social inequalities in health. The HCSP analyzed, on the one hand, the combined effect of numerous determinants on the COVID-19 epidemic and its evolution within the population and, on the other hand, the effects of the crisis on socio-economic determinants, social inequalities in health and the health status of the population. In this opinion, the HCSP recommends that "any health crisis, including infectious ones, should be considered as a syndemic crisis”.

## Method

A public health conceptual framework adapted to the COVID-19 health crisis was developed; it was based on a global approach to health, as the evolution of the epidemic is, by definition, multifactorial. To build it, the HCSP used two approaches, one graphical and the other analytical [8].

- The graphic approach, or concept map, aims to provide a common understanding of all the fields and domains that influence the evolution of the SARS-CoV-2 epidemic and are identified as determinants of health [6].
- This conceptual framework allows for the identification of synergies and multidisciplinary interactions, the incorporation of isolated initiatives into an integrative framework, the formalization of strategies and the implementation of cross-cutting and systemic actions on population health.
- The analytical approach makes the conceptual scheme functional. It is broken down into several interactive technical sheets. These sheets specify the diagnoses based on the predefined determinants and identify the points of vigilance and alert. Based on the findings of the health crisis assessment and the points of vigilance and alert identified in these 10 sheets, the HCSP proposes recommendations for anticipating the consequences of a pandemic crisis on the health of the population and for developing strategies to reduce them.

### Graphic approach and conceptual public health framework

Many conceptual models in public health have been developed, in particular L. Green's PRECEDE- PROCEED [9, 10].

Well known to health promotion practitioners, this planning model is based on the disciplines of epidemiology, social and behavioral sciences and education. The principles behind the construction of this model stem from the multi-factorial nature of any problem. As a corollary, public health policies, programs and actions, in order to act on behavior, environment and social factors, are necessarily multidimensional and multisectoral.

The acronym PRECEDE stands for "Predisposing, Reinforcing and Enabling Constructs in Educational/Environment Diagnosis and Evaluation" or "the predisposing, facilitating and reinforcing factors identified by the educational and environmental diagnosis and evaluation of this diagnosis" [11].

The acronym PROCEED stands for "Policy, Regulatory and Organizational Contructs in Educational and Environmental Development". This system approach model is based on different diagnoses: epidemiological, behavioral, environmental, educational, organizational and administrative. Any PRECEDE-PROCEED model is built on the basis of available data and can be enriched by the experiences of the different contributors.

### Analytical and foresight approach

From a search for certainties about the future (forecasting), and in response to the growing complexity and uncertainties of subjects and problematics, futures studies have moved to a more integrated, complex and diachronic way of thinking the future [12].

For a group of expert of different fields, integrating foresight requires to discuss a common Foresight Framework, defined as the combination of the worldview, the analytical grid and the foresight lens [13].

One of the pioneers in France, Gaston Berger [14] sets out the six criteria of foresight:

- to see far into the future: to place phenomena in an evolutionary perspective;
- to see broadly: to take into account all possible perspectives, human, geographical, political, etc.;
- to analyze in depth: to detect the structural trends that guide evolution;
- thinking together: because a single brain is no longer capable of grasping a phenomenon in all its complexity, for the benefit of the greatest number (collective intelligence);
- taking risks: making choices by appreciating the global impact of our actions;
- thinking about people: action is only worthwhile if we work for people and not against them (the sense of humanity).

Beyond the multiple tools and methods of foresight, a "meta-method" of foresight, transverse to all other methods, structures the approach [13]. This method has the merit of simplicity and the ability to integrate all the tools of French and foreign foresight. It comprises three distinct cognitive stages: understanding, anticipation and proposals of action. Each of them has its own characteristics in thinking about the future and can produce a specific type of study (such as a diagnosis for understanding, a scenario for anticipation, a strategic analysis for the proposal for action). But it is the integrated process—in its entirety—that gives meaning to prospective thinking.

**Fig. 1:**
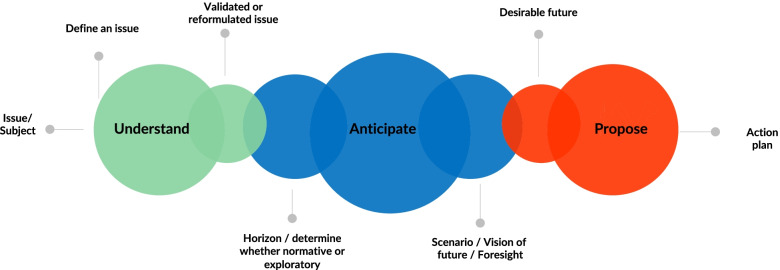
From attitude to prospective action: a meta-method scheme (Adapted from Goux-Gaudiment, 2014)

The most standard operating mode consists of analyzing the elements of understanding of the object studied from different angles, then bringing them together into a systemic vision that obeys the characteristics of foresight (UNDERSTAND); then imagining the possible evolutions of this system according to internal or external factors of change (ANTICIPATE); and finally choosing a vision of the future from among several desirable scenarios and proposing—to implement it—actions that create or correct change, adapted to the reality of the present situations and to the situations anticipated in the short, medium and long terms (PROPOSE) (Fig. 1).

### Graphic approach: designing a systemic scheme adapted to the pandemic

**Fig. 2:**
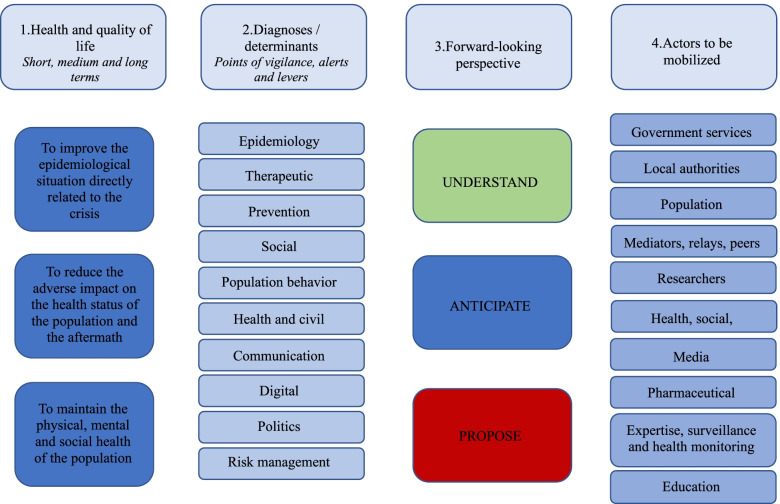
High Council for Public Health (HCSP) conceptual scheme adapted for Covid-19 pandemic for public health planning based on L. Green's model (Green and Hreuter, 1991 and 1999). Arrows illustrate the link and interactivity between variables in columns and lines

In order to understand the pandemic crisis in a forward-looking way, the HCSP has designed a conceptual scheme for public health planning based on L. Green's model (Fig. 2). It sets out short, medium and long-term objectives (Fig. 2, column 1): (i). Improve the epidemiological situation directly related to the epidemic; (ii). Preserve the physical, mental and social health of the population; (iii). Avoiding adverse spillover/deleterious side effects of preventive strategies on health status and sequelae.

To illustrate the multifactorial nature of the problem, 10 areas covering all the individual, environmental, social and organizational determinants that impact on the objectives were identified by the working group of the HCSP. A specific diagnosis (Fig. 2, Column 2) was drawn up for each of them; this made it possible to identify the points of vigilance and alert on the one hand and the levers on the other. In a forward-looking vision, the whole process is broken down into three stages (Fig. 2, Column 3): understanding the problem, anticipating a scenario, and proposing a roadmap that includes operational objectives formulated in the form of precise measures. Finally, all the professionals, structures, bodies and experts to be mobilized are identified (Fig. 2, Column 4). The interactions between the components of the plan reflect the complexity of managing an epidemic health situation.

### Analytical approach: construction of 10 technical sheets and recommendations

**Fig. 3:**
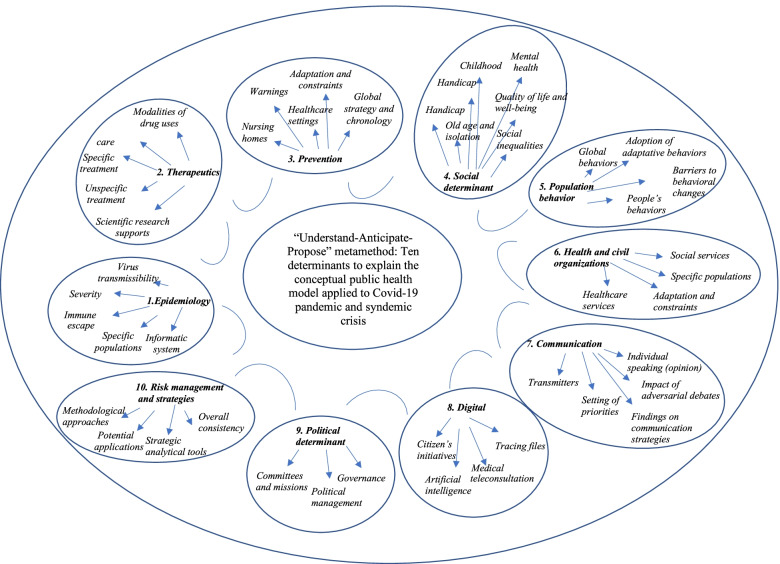
Essential diagnoses and integrating different determinants used to analyze the pandemic and syndemic Covid-19 crisis and to provide recommendations

The conceptual framework is broken down into 10 technical sheets (Fig. 3) covering essential diagnoses and integrating all the determinants. Based on the state of knowledge at the time of writing, they define the warning points and the levers for action in the following fields.

Each of these was broken down into three cognitive stages, allowing for a diagnosis of understanding, a scenario of anticipation and a strategic analysis of action according to the chronology: understand-anticipate-propose. From these 10 technical sheets, 32 recommendations have been proposed through 10 specifics axes to ease appropriation by the politics (Table 1).

**Table 1:** HCSP Conceptual framework broken down into 10 axes covering essential diagnoses and integrating all the determinants and recommendations

Axes	Recommendations
**1.** Syndemic nature of Covid-19 pandemics	**R1.** Measure the impact of pandemics and control measures on the health status of the general population (co-morbidity, mental health)
	**R2.** Take into account the societal effects of pandemics and prevent the aggravation of social inequalities in health (SSI)
**2.** Health promotion / Prevention	**R3.** Develop confidence and acceptance in the general population
	**R4.** Deploy Health Education programs (individual reflection) in schools and in the general population
	**R5.** Strengthen the training of front-line professionals (healthcare setting (ES), nursing homes (ESMS), national education, occupational health, associations, local authorities, etc.)
	**R6.** Ensure the availability of barrier measures and the management of screening in the general population
	**R7.** Promote vaccination
**3.** Social debate in the management of pandemics	**R8.** Organize individual (community approach) and institutional (WHO health democracy) societal participation (WHO, 2020)
	**R9.** Integrate and take into account the contributions of human and social science (SHS) experts in scientific committees for health policy decision-making
	**R10.** Better understanding of vaccine hesitancy (5C model) (SAGE, 2014) [15]
**4.** Pandemic management based on risk and uncertainty reduction strategies	**R11.** Define individual and collective objectives of strategic control measures in advance
	**R12**. Use risk analysis and foresight methods
	**R13.** Monitor good practice in impact assessment of public interventions. Develop a risk reduction approach to crisis management, taking into account the differentiated impact of measures on people's overall health
**5.** Availability of national epidemiological databases for pandemic management	**R14.** Make available and facilitate the use of national databases (regulatory aspect and interoperability)
	**R15.** Promote research and industrial logistics to adapt vaccine and therapeutic strategies during a pandemic
	**R16.** Consider alert systems that are better adapted to serious and rare diseases in children
**6.** Concerted and international scientific research into medicines	**R17.** Rapidly define priority questions in a concerted manner at national, European and international levels
	**R18.** Provide the means to respond rapidly to priority research areas using scientific protocols that meet quality criteria defined in consultation with methodologists
**7.** Interdisciplinary intervention research (public health, social sciences and humanities) on behavior and its environment	**R19.** Develop multidisciplinary research programs on social factors, financing systems, organizational structures and processes, health technologies and personal behavior in accordance with the guidelines of the Medical Research Council for the management of global health trials (MRC, 2021) -Improve knowledge of target populations at risk of severe disease -Identify and prevent the impacts of a pandemic crisis on specific populations (e.g. disability, children, elderly people, etc.)
	**R20.** Test pandemic control strategies (attitude—knowledge—behavior) -Improve knowledge on individual and collective adherence to control measures -Understand factors related to particular populations
	**R21.** Assess the impact of the information crisis in the health crisis
	**R22.** Promote Health services research. Research areas are individuals, families, organizations, institutions, communities and populations (Lohr and Steinwachs, 2002)
**8.** Professionalized communication	**R23.** Define specific strategies according to the political (decision) or scientific (knowledge) sender
	**R24.** Integrate the ethical dimension and deontological imperatives into communication strategies
	**R25.** Set up and provide training in public speaking and media training for professionals who have to speak in the context of a pandemic and officially represent their institution
**9.** Innovation and digital	**R26.** Ensure a scientific watch on innovations in terms of improving patient care and the quality of life of the general population
	**R27.** Guarantee or facilitate access to digital services for all
	**R28.** Reduce social inequalities in health through the democratization of innovations and digital technology
	**R29.** Ensure the security (cyber security) of health data and information systems
**10.** Health and civil organizations	**R30.** Draw up business continuity plans and white plans that are as realistic as possible and adapted to pandemic situations
	**R31**. Provide schools and universities with public health resources to deal with possible new epidemic episodes (help with prevention and screening, etc*.*)
	**R32.** Develop training in contingency planning and cooperation for staff in organizations that will be managing responses to future health crises

## Discussion

In April 2021, the HCSP set up an "Evaluation, Strategy, Foresight" working group to consider the future of the pandemic crisis and its systemic consequences on society, beyond the infectious aspect alone. The objective of this work was to define a conceptual model of public health that could take into account all the determinants and dimensions of this crisis in order to better understand the issues at stake, by identifying dangers and levers for action. The aim was to establish priorities based on the many recommendations made in order to anticipate the collateral consequences of the management of the crisis and better prepare for the next one. Foresight and scenario analysis exercise will follow this initial work.

The priorities identified below will be articulated through various technical and operational guidelines. To meet this objective, the HCSP working group used a "Understand—Anticipate—Propose" meta-method.

From the beginning of the management of the pandemic, the relative absence of a global, formalized strategic framework, discussed between the various stakeholders in the decision-making process for the public authorities, and communicated in an appropriate manner to the public, contributed to the many questions raised by society, particularly those related to the inconsistencies (supposed, real or perceived) of the measures taken. A strategic approach could have helped to better verify and explain the choice of certain measures (e.g. that the most cost-effective measures are favored, which may lead to different actions in apparently similar contexts).

The crisis we have been experiencing since January 2020 has understandably been seen as an infectious disease to be controlled. Initially, therefore, all interventions focused on controlling the viral transmission of SARS-CoV-2, thereby controlling the spread of the pathogen. The "science" that has guided governments has been conducted primarily by epidemic modelers and infectious disease specialists. But what we have learned so far indicates that the COVID-19 story is not so simple, because in addition to the COVID-19 patients, non-infectious disease patients must be considered. These two types of patients are grouped together in social groups according to patterns of inequality that are deeply rooted in our societies [3].

Thus, COVID-19 is not just a pandemic. It is a syndemic crisis. The syndemic nature of the threat we face means that a more nuanced approach is needed to protect the health of the general population. What characterizes this pandemic, then, is its syndemic nature. Syndemia, a concept coined by Merill Singer [2] could be used to highlight this breakdown in equality and help decompartmentalize health, economic and social policies. Defined as "the aggregation of two or more diseases or health problems in a population for which there is some level of deleterious biological or behavioral interface that exacerbates the negative effects of each of the diseases involved", syndemics are characterized by interactions between socio-economic conditions and health status.

The syndromic model of health focuses on the biosocial complex, which consists of interacting, co-presenting or sequential diseases and the social and environmental factors that promote and reinforce the negative effects of the interaction of diseases. This emerging approach to the conception of health and clinical practice reconfigures the conventional historical understanding of diseases as distinct entities in nature, separate from other diseases and independent of the social contexts in which they occur. Instead, all of these factors tend to interact synergistically, in various ways and in a consequential manner, having a substantial impact on the health of individuals and whole populations. Specifically, a syndemic approach examines the reasons why certain diseases cluster (i.e. multiple diseases affecting individuals and groups); the pathways by which they interact biologically in individuals and within populations, thereby multiplying their overall burden of disease; and the ways in which social environments, particularly conditions of social inequality and injustice, contribute to disease clustering and interaction and to vulnerability.

These interactions are intertwined and reinforced particularly for certain social groups. The risk of a deterioration in their health status and socio-economic conditions increases. This notion invites us to imagine another form of policy, one that is likely to go beyond the sole response to the pandemic. For the socially unequal impact of COVID-19 is not limited to infection and mortality from the virus. It is also characterized by the health, social and economic consequences of the solutions proposed in most countries to try to limit infection. The collateral damage of measures such as containment also follows a social gradient. Both in the short term—with different experiences of containment, notably according to the quality of the place of living, the permanence of employment and income, the possibility or not of teleworking, or access to a green space—and in the longer term, with the economic and social consequences of "high containment".

Health is not just an individual issue or a matter of personal data (health check-ups, analyses, etc.). According to Singer et al. "A syndemic approach offers a very different direction for clinical medicine and public health by showing how an integrated approach to understanding and treating diseases can be much more effective than simply controlling an epidemic disease or treating patients individually” [2].

In recent times, the COVID-19 crisis has shown how all these layers are intertwined and how they need to be thought of together to deal with the pandemic. The notion of syndromes is an interesting way of accounting for this complexity. Managing this crisis solely from a health perspective, or even from the perspective of co-morbidity (hypertension, obesity, diabetes, chronic cardiovascular and respiratory diseases, as well as cancerous pathologies) limits its effectiveness.

Indeed, these non-communicable diseases are distributed in the population according to a social gradient inversely proportional to wealth. In other words, their prevalence increases as the economic and social capital of individual decreases. This social gradient also illustrates the notion of syndromes: people who are economically fragile and have multiple co-morbidities are those who have paid the highest price for COVID-19 and its management.

Green's planning scheme makes it possible to present the potential determinants of health problem and above all to provide elements of a response. The ultimate element, on which we hope to act (in the long term), is collective health, taking into account the strong variations in individual objectives of quality of life and perception of health risks. Two main factors impact on quality of life: individual behavior and lifestyle on the one hand, and living environments on the other. It is very difficult to have a direct influence on them and it is therefore necessary to act upstream, on the factors on which they depend:

- predisposing factors: preconditions for change (knowledge, attitudes, beliefs, values, habits, etc.);
- facilitating factors: what in the living environment makes it easier or harder to adopt behavior (structures, individual and collective resources, etc.);
- reinforcing or hindering factors: gratification or punishment (peer opinion, media, reference images, pleasure or displeasure caused by a behavior, etc.).

The conceptual model of public health, inspired by Green and created by the HCSP, attempts in this article to understand the stakes of such a crisis on the basis of determinants, to define the dangers and levers that would make it possible to anticipate a future pandemic, and finally, within the framework of an action plan, to propose the main lines of thought and actions. The objective of this conceptual and functional model is the health and well-being of the general population, in accordance with the WHO definition [16].

For the WHO, health is a "state of complete physical, mental and social well-being and not merely the absence of disease or infirmity". This is why it is now important to put this holistic approach to health back at the heart of the syndemic management strategy, which cannot be reduced to the absence or presence of disease alone. In other words, beyond the infectious aspect, it affects a great many pathologies, particularly chronic diseases, which pose the problem of their management within the framework of deprogramming and that of being risk factors for infection by SARS-CoV-2. It is also a societal crisis with an economic and social upheaval which of course entails a risk of aggravating social inequalities in health, but also poses the question of how to manage the pandemic [15], and the current difficulties with vaccination and the various prevention measures reflect the extremely deep social aspect of prevention issues [17].

HCSP proposes numerous operational recommendations stratified within different axes of analysis of the pandemic crisis. These different axes illustrate perfectly the syndemic character of the crisis and propose to political decision-makers a range of measures that could help anticipate a future crisis or prevent it from becoming chronic.

These 10 areas of recommendations were built from the analysis of the different determinants as well as the associated dangers and levers of action. They correspond to approaches related to the health system (axes 1 and 2), to crisis management (axes 3–5 and 10), to research and innovation (6, 7 and 9) and finally to professional communication (axis 8). Taking these recommendations into account in terms of health policy strategy should allow for an approach that is less focused solely on the aspect of medical care, and allow for the anticipation and management of the other consequences of the crisis (social, economic, mental health, digital, etc.).

## Conclusion

The public health model created by the HCSP and inspired by Green's conceptual model attempts in this article to understand the stakes of this syndemic crisis based on different determinants. This work is intended to allow reflections on public health approaches to strengthen and anticipate health crisis management and health planning by politic managers working at national or sub-national level.

## Data Availability

All data and materials used in the writing are described in the manuscript and are available and no additional data exist.

## References

[1] World Health Organization (2004). Les Déterminants sociaux de la santé: Les Faits , 2004.

[2] Singer M, Bulled N, Ostrach B, Mendenhall E (2017). Syndemics and the biosocial conception of health. Lancet.

[3] Horton R (2020). Offline: COVID-19 is not a pandemic. Lancet.

[4] High Council for Public Health (2021). Crise sanitaire de Covid-19 et inégalités sociales de santé.

[5] Whitmee S, Haines A, Beyrer C, Boltz F, Capon AG, Ferreira de Souza Dias B (2015). Safeguarding human health in the Anthropocene epoch : report of The Rockefeller Foundation-Lancet Commission on Planetary Health. Lancet.

[6] World Health Organization. Health Promotion Glossary, Health Promotion and Communication Division, Health Education and Promotion Department, Geneva; 1999. p. 25 http://whqlibdoc.who.int/hq/1998/WHO_HPR_HEP_98.1_fre.pdf

[7] World Health Organization. Constitution of the World Health Organization, www.who.int/governance/eb/who_constitution_fr.pdf

[8] Haut Conseil de la santé publique. Guidelines on forward thinking and points of vigilance in times of health crisis, https://www.hcsp.fr/Explore.cgi/avisrapportsdomaine?clefr=1143

[9] Green LW, Kreuter MW (1991). Health Promotion Planning: An Educational and Environmental Approach.

[10] Green LW and Kreuter MW. Mountain View, CA: Mayfield Publishing Company, 1999; 621 pp. Health Promotion Planning: An Educational and Ecological Approach (3rd ed). Can J Public Health. 2001; 92(5):384.

[11] Renaud L (2020). Planifier pour mieux agir.

[12] Goux-Baudiment F (2016). A Foresight Overarching Method: I. Looking for a Way to Bridge the Gap. World Futures Rev.

[13] Goux-Baudiment F, Brunet Sébastien, Guyot Jean-Luc (2014). « De l’attitude à l’action prospective : une méta-méthode* »*. Construire les futurs – Contributions épistémologiques et méthodologiques à la démarche prospective.

[14] Berger G (1957). La Revue des Deux Mondes.

[15] World Health Organization (2020). The health democracy deficit and Covid-19, 2020.

[16] World Health Organization (2020). Regional Office for E. Pandemic fatigue: reinvigorating the public to prevent COVID-19: policy framework for supporting pandemic prevention and management: revised version November 2020.

[17] The SAGE Vaccine Hesitancy Working Group (2014). Report of the SAGE working group on vaccine hesitancy.

